# Costs of Prescription Drugs for Children and Parental Adherence to Long-Term Medications

**DOI:** 10.1001/jamanetworkopen.2023.37971

**Published:** 2023-10-16

**Authors:** Julie C. Lauffenburger, Renee A. Barlev, Eniola Olatunji, Gregory Brill, Niteesh K. Choudhry

**Affiliations:** 1 Division of Pharmacoepidemiology and Pharmacoeconomics, Department of Medicine, Brigham and Women’s Hospital and Harvard Medical School, Boston, Massachusetts; 2 Center for Healthcare Delivery Sciences (C4HDS), Department of Medicine, Brigham and Women’s Hospital and Harvard Medical School, Boston, Massachusetts; 3 Now with Vytalize Health

## Abstract

**Importance:**

The adverse effects of prescription drug costs on medication adherence and health have been well described for individuals. Because many families share financial resources, high medication costs for one could lead to cost-related nonadherence in another; however, these family-level spillover effects have not been explored.

**Objective:**

To evaluate whether the cost of a child’s newly initiated medication was associated with changes in their parent’s adherence to their own medications and whether that differed by likely duration of treatment.

**Design, Setting, and Participants:**

This cohort study used interrupted time-series analysis with a propensity score-matched control group from a large national US health insurer database (2010-2020) and included children initiating medication and their linked presumed parents using long-term medications.

**Exposure:**

The cost of the child’s initiated medication. Child medication cost was classified based on highest (≥90th) or lowest (<10th) decile from out-of-pocket medication spending, stratified by whether the medication was intended for short- or long-term use. Children initiating high-cost medications (based on the highest decile) were propensity-score matched with children initiating low-cost medications.

**Main Outcome and Measures:**

The child’s parent’s adherence to long-term medication assessed by the widely used proportion of days covered metric in 30-day increments before and after the child’s first fill date. Parent demographic characteristics, baseline adherence, and length of treatment, and family unit size and out-of-pocket medication spending were key subgroups.

**Results:**

Across 47 154 included pairs, the parents’ mean (SD) age was 42.8 (7.7) years. Compared with a low-cost medication, initiating a high-cost, long-term medication was associated with an immediate 1.9% (95% CI, −3.8% to −0.9%) reduction in parental adherence sustained over time (0.2%; 95% CI, −0.1% to 0.4%). Similar results were observed for short-term medications (0.6% immediate change; 95% CI, −1.3% to −0.01%). Previously adherent parents, parents using treatment for longer periods, and families who spent more out-of-pocket on medications were more sensitive to high costs, with immediate adherence reductions of 2.8% (95% CI, −4.9% to −0.6%), 2.7% (95% CI, −4.7% to −0.7%), and −3.8% (95% CI, −7.2% to −0.5%), respectively, after long-term medication initiation.

**Conclusions and Relevance:**

In this cohort study small reductions in adherence across parents with higher child drug costs were observed. Health care systems should consider child-level or even household-level spending in adherence interventions or prescription policy design.

## Key Points

**Question:**

Although individual cost-related nonadherence has been studied extensively, what is the association between high prescription drug costs and adherence to medications for long-term conditions across families?

**Findings:**

In this cohort study with 47 154 child-parent pairs, high prescription costs by children for newly initiated medications was associated with small, but sustained, reductions in their parents’ long-term adherence to medication; families who spent more out-of-pocket on medications and parents who were previously adherent were more sensitive to high prescription costs.

**Meaning:**

The findings of this study suggest that health care systems should address children or household-level medication spending in adherence intervention or policy design.

## Teaser

This cohort study examines whether the cost of a newly initiated medication by a child affected their parent’s adherence to their own existing medications and whether that differed by likely duration of treatment.

## Introduction

High prescription drug costs contribute to poor adherence to medication, leading to adverse downstream effects on health care spending and health outcomes.^1,2,3,4,5,6,7^ The relationship between high drug costs and nonadherence has been well described for individuals.^2,8,9,10,11^ However, many families share financial resources, so high medication costs for one could lead to cost-related nonadherence in another.^12,13^ This relationship may also explain high correlations in nonadherence between adult family members previously observed.^14,15^ To our knowledge, spillover effects of cost-related nonadherence on others in families have not been explored.

One way that cost-related nonadherence might manifest within families is high prescription costs among children and adolescents. Spending for prescription drugs for children has increased substantially over the past several decades^16,17,18,19,20,21,22^; and higher numbers are using medication than ever before.^20,21,22,23^ For example, more than 20% of children used a prescription drug in the past month,^23^ including those for long-term conditions, like diabetes or asthma.^20^ Low-income child and adolescent beneficiaries, even those funded by employer plans, have even higher rates of prescription use.^24^ The effect of prescription costs may also vary based on likely duration of treatment, such as for short- or long-term use, or other factors, such as how long the family member has been using treatment.

We hypothesized that high medication costs for one family member could lead to cost-related nonadherence in another. Therefore, we sought to evaluate whether the cost of a newly initiated medication by a child affected their parent’s adherence to their own existing medications and whether that differed by likely duration of treatment.

## Methods

### Data Source

This cohort study used an interrupted time-series analysis using claims for commercially-insured individuals receiving benefits from Optum (deidentified Clinformatics Data Mart Database), a large health insurer covering patients in all 50 states in the US. We used deidentified patient-level claims for inpatient and outpatient procedures, hospitalizations, emergency department and office visits, and outpatient prescription dispensations linked with plan enrollment data from 2010 to 2020. Identifiers link members of a “family unit” based on a shared subscriber number within a group/policy, uniquely identified in the insurer. Because these identifiers are used for policy coverage decisions, the identifiers are considered reliable. The Mass General Brigham institutional review board approved this study and approved a waiver of consent because all data were deidentified. We followed the Strengthening the Reporting of Observational Studies in Epidemiology (STROBE) reporting guidelines.^25^

### Study Population

A schematic is shown in eFigure 1 in Supplement 1. We created a list of the top 200 prescription drugs initiated by individuals younger than 18 years to ensure representativeness and also classify medications (covered >95% of fills). The date that the child first filled an eligible medication during the study period was defined as their index date. We defined initiation as not filling 1 of these medications before the index date. To be included, children had to have 360 or more days of continuous enrollment before the index date and have nonmissing sex and age. Each child’s first eligible index date was selected; and, if the child filled more than 1 medication on the index date, 1 was randomly chosen. Medications were classified as short term or long-term based on whether they were written as a 1-time prescription (eg, amoxicillin) or intended for long-term use (eg, topiramate), stratifying them into short-term and long-term cohorts.^26^

Using the family identifier, we then identified parents of these children taking a long-term medication on their child’s index date (ie, filled in the past 360 days). Consistent with prior approaches,^14,15^ presumed parents were defined as individuals in the same family who were aged 15 or more years older than the child and aged 18 years or older. The list of medications were generated from the 200 most commonly-filled medications by adults (covered >90% of fills). From this list, we excluded medications used episodically (antibiotics, estrogen and/or progesterone, depression and/or anxiety medications) and liquid and/or injectable medications owing to difficulties calculating adherence. To be included, parents also had to have had continuous insurance enrollment in the 360 days before the index date. If parents had multiple eligible medications, the medication with a fill closest to the child’s index date was selected to avoid assumptions about multiple medication use^27^; if multiple fills were eligible on the same day, 1 was randomly selected. If more than 1 child within the same family unit met criteria, the child with the earliest index date was selected. If the included child had more than 1 eligible parent, 1 was randomly selected for inclusion to minimize correlations within the data (and only approximately 13% had multiple eligible parents, eTable 1 in Supplement 1). Thus, each child, parent, pair, and family unit was only included once across the cohorts.

### Exposure

The primary exposure was the out-of-pocket cost of the medication initiated by the child in each child-parent pair. Out-of-pocket costs were calculated based on the amounts of all copayments, coinsurance, and deductible on the index prescription. For each cohort (long term and short term), we classified initiated medications in the highest decile (≥90%) of out-of-pocket costs on the index claim as being high cost and those in the lowest decile (<10%) of costs as being low cost and retained those individuals for the analysis. Alternative definitions were used for sensitivity analyses.

### Outcomes

The primary outcome was change in adherence in the presumed parent of each eligible child. Adherence was assessed by applying the proportion of days covered (PDC) metric to pharmacy claims data, a standard quality measure.^27,28,29^ To measure PDC, we generated a supply diary for the parent’s medication by aggregating consecutive fills based on dispensing date and days supplied, starting 539 days before the child’s index date (or the parent’s first fill before that date) through 360 days after the index date.^11^ We allowed supply to accumulate to 180 days; days spent in a facility were considered covered days. Parents were censored on loss of enrollment.

Using this supply diary, we calculated PDC within each month (normalized to 30 days) from 360 days before the index date to 360 days after (24 months total). Overall, PDC was calculated by dividing the number of days with medication available each month by the number of days the parent contributed that month. We measured PDC as both a continuous (primary outcome) and secondary dichotomous adherence measure, with PDC of 80% or greater considered optimal.^27,30,31^

### Covariate Assessment

We measured several baseline characteristics of the child, parent, and family unit (all within the same family identifier) (Table 1). In both child and parent, we measured age, sex, database-reported algorithm of race and ethnicity, whether the study medication was branded or acquired by mail order, and number of unique medications filled in the baseline period by generic name directly from claims. For parents, we also measured region of residence, including state, number of physician office visits and hospitalization days, comorbidities, and combined comorbidity score.^32,33,34^ Comorbidities were defined using *International Classification of Diseases, Ninth Revision (ICD-9)* or *ICD-10* codes, as applicable, using codes defined by the Centers for Medicare & Medicaid Services Chronic Condition Warehouse.^35^ Within each family unit, we measured the total number of children (aged <18 years on the index date) and prior year out-of-pocket spending on pharmacy and medical costs (excluding the child’s index medication).

**Table 1.** Baseline Characteristics for High-Cost and Low-Cost Child-Parent Pairs

Characteristic	Pairs in the long-term cohort, No. (%)		ASD^a^	Pairs in the short-term cohort, No. (%)		ASD^a^
	Lowest decile (n = 2387)	Highest decile (n = 2387)		Lowest decile (n = 21 190)	Highest decile (n = 21 190)	
Child						
Age, mean (SD), y	12.6 (5.0)	12.6 (3.7)	0	9.7 (5.4)	9.8 (4.8)	0
Sex						
Female	1519 (63.6)	1525 (63.4)	0.01	10 638 (50.2)	10 618 (50.1)	0
Male	868 (36.4)	862 (36.6)	0.10	10 552 (49.8)	10 572 (49.9)	0
Mail order for study medication	26 (1.1)	30 (1.3)	0.02	8 (0.1)	29 (0.1)	0.03
Unique No. of medications, mean (SD)	1.3 (0.7)	1.2 (0.6)	0.03	1.3 (0.6)	1.3 (0.6)	0
Parent						
Age, mean (SD), y	44.9 (7.8)	45.0 (7.4)	0	42.5 (7.8)	42.6 (7.6)	0
Sex						
Female	1179 (49.4)	1180 (49.4)	0	10 236 (48.3)	10 220 (48.2)	0
Male	1208 (50.6)	1207 (50.6)	0	10 954 (51.7)	10 970 (51.8)	0
Race and ethnicity						
Asian	113 (4.7)	122 (5.1)	0.02	1487 (7.0)	1449 (6.8)	0.01
Black, non-Hispanic	198 (8.3)	184 (7.7)	0.02	1652 (7.8)	1643 (7.8)	0
Hispanic	213 (8.9)	211 (8.8)	0	2311 (10.9)	2274 (10.7)	0.01
White	1652 (69.2)	1655 (69.3)	0	13 855 (65.4)	13 919 (65.6)	0
Other/unknown	211 (9.3)	215 (9.0)	0.01	1885 (8.9)	1905 (9.0)	0
Region						
Northeast	221 (9.3)	144 (6.0)	0.12	1283 (6.1)	1431 (6.8)	0.03
Midwest	436 (18.3)	470 (19.7)	0.04	3871 (18.3)	3947 (18.6)	0.01
South	626 (26.2)	606 (25.4)	0.02	5984 (28.2)	5969 (28.2)	0
West	328 (13.7)	334 (14.0)	0.01	2823 (13.3)	2831 (13.4)	0
Other/unknown	776 (32.5)	833 (34.9)	0.05	7229 (34.1)	7012 (33.1)	0.02
Medication characteristics						
Mail order for study medication	114 (4.8)	125 (5.2)	0.02	1002 (4.7)	1019 (4.8)	0
Brand name for study medication	175 (7.3)	185 (7.8)	0.02	1683 (7.9)	1704 (8.0)	0
Health resource utilization, mean (SD)						
Unique No. of medications	5.6 (4.2)	5.6 (4.3)	0	5.8 (4.2)	5.8 (4.1)	0
Physician office visits	4.8 (4.4)	4.8 (4.4)	0	5.0 (4.6)	5.0 (4.5)	0
Days hospitalized	0.5 (3.8)	0.4 (2.6)	0	0.4 (3.8)	0.4 (2.6)	0
Comorbidities						
Combined comorbidity score, mean (SD)	0.1 (1.0)	0.2 (1.1)	0.02	0.2 (1.1)	0.2 (1.1)	0
Acquired hypothyroidism	239 (10.0)	239 (10.0)	0	2568 (12.1)	2535 (12.0)	0
Asthma	80 (3.4)	85 (3.6)	0.01	826 (3.9)	854 (4.0)	0.01
Atrial fibrillation	19 (0.8)	22 (0.9)	0.01	148 (0.7)	150 (0.7)	0
Chronic kidney disease	66 (2.8)	69 (2.9)	0.01	659 (3.1)	668 (3.2)	0
COPD	12 (0.5)	19 (0.8)	0.04	135 (0.6)	134 (0.6)	0
Depression	303 (12.7)	310 (13.0)	0.01	2511 (11.9)	2558 (12.1)	0.01
Diabetes	211 (8.8)	216 (9.1)	0.01	1986 (9.4)	1973 (9.3)	0
Epilepsy	12 (0.5)	15 (0.6)	0.02	109 (0.5)	114 (0.5)	0
Heart failure	16 (0.7)	23 (1.0)	0.03	189 (0.9)	180 (0.9)	0
Hyperlipidemia	506 (21.2)	490 (20.5)	0.02	4240 (20.0)	4228 (20.0)	0
Hypertension	592 (24.8)	588 (24.6)	0	5007 (23.6)	5024 (23.7)	0
Ischemic heart disease	93 (3.9)	92 (3.9)	0	686 (3.2)	666 (3.1)	0.01
Liver disease	26 (1.1)	22 (0.9)	0.02	279 (1.3)	276 (1.3)	0
Stroke/transient ischemic attack	23 (0.9)	21 (0.9)	0.01	167 (0.8)	184 (0.9)	0.01
Family unit, mean (SD)						
Total No. of children	2.05 (1.1)	2.1 (1.0)	0.01	2.1 (1.1)	2.1 (1.0)	0.01
Total family OOP pharmacy costs,^b^ $	686.4 (1148.8)	741.0 (876.8)	0	601.1 (1001.6)	623.4 (726.6)	0
Total family OOP medical costs, $	5849.0 (6582.9)	5787.7 (6277.4)	0	5667.2 (6648.4)	5611.2 (6521.9)	0

Abbreviations: ASD, absolute standardized difference; COPD, chronic obstructive pulmonary disease; OOP, out-of-pocket.

^a^ ASD lower than 0.1 was considered to indicate good balance, data shown after propensity score matching.

^b^ Excluding cost of study drug.

### Statistical Analysis

We used an interrupted time-series approach^5,11,36^ to evaluate the association between the cost of the child’s initiated medication on their parent’s adherence, comparing those initiating high-cost vs low-cost medications, and conducted analyses separately for child short-term and long-term medications. We used propensity score matching separately in each cohort. Specifically, we calculated the propensity to initiate a high-cost medication among those in the high decile vs the low decile, incorporating all baseline characteristics in Table 1 and PDC measured in the month before the child’s index date, to further account for baseline trends.^37,38^ We then used greedy matching (5:1 digit matching) and 1:1 sampling without replacement excluding those with nonoverlapping scores,^38,39,40^ evaluating balance through postmatching absolute standardized differences lower than 0.1 and C statistics close to 0.5 (ie, little ability to discriminate after matching).^41,42^

Within these matched cohorts, we plotted monthly trends in the parent’s medication adherence in the 12 months before and after their child’s index date. We then used generalized estimating equations with an exchangeable correlation structure to adjust for repeated measurements of adherence for each individual. The model included variables for the exposure (high-cost), month (1-24), a prepost index date indicator (months 1-12 or 13-24), time in months since the index date, interaction term between the exposure and postindex date indicator, and interaction term between exposure and time in months since the index date. These variables measure the immediate change (level) and subsequent trend (slope) in adherence after the child’s index date. For the continuous PDC outcome, we used an identity link function with normally-distributed errors; for the secondary dichotomous PDC outcome, we used a log-link function with Poisson-distributed errors. We conducted analyses separately for short-term and long-term cohorts.

We conducted several exploratory subgroup analyses including (1) parent sex, (2) parent age, (3) parent race and ethnicity, (4) optimal adherence for the parent at baseline (PDC ≥80%), (5) whether the parent recently started the medication (initiating in the <180 days prior to the child’s index date), (6) whether the family unit was in the top 25% of prior year out-of-pocket spending on pharmacy costs (excluding child index drug cost), and (7) if the family unit had more than 1 child. We also conducted sensitivity analyses of definitions and modeling assumptions. These included (1) defining high cost and low cost by quartiles and above and below the median rather than deciles, (2) restricting to child-parent pairs with 20 or more years’ age difference,^14^ (3) including a 90-day transition period to allow for the potential for a lagged effect (particularly for medications with longer days’ supplied), and (4) extending the exposure window to include the full first 90 days of spending on the index drug to define spending (and restarting follow-up). Analyses were conducted using SAS statistical software (version 9.4; SAS Institute, Inc) with a 2-sided *P* < .05 as the significance level. Analyses were conducted between June 2021 and August 2022.

## Results

In total, 4774 and 42 380 child-parent pairs were included in the long-term and short-term cohorts (eTable 1 in Supplement 1). Baseline characteristics before and after matching are in eTable 2 in Supplement 1 and Table 1, respectively. Postmatch C statistics were 0.54 and 0.54 for the long-term and short-term cohorts, indicating good balance. The only imbalanced covariate after matching was Northeast region in the long-term cohort.^41^ As shown in Table 1, presumed parents’ mean (SD) ages were 42.5 (7.8) years in the short-term cohort and 44.9 (7.6) years in the long-term cohort.

The median (IQR) out-of-pocket child’s costs for the matched long-term high-cost and low-cost medication groups were $133.79 ($90.00-$188.49) and $0 ($0-$0), respectively. For the short-term high-cost and low-cost groups, median (IQR) out-of-pocket costs were $50.00 ($40.00-$74.00) and $2.15 ($0-$3.32). Parents’ baseline spending is shown in eTable 3 in Supplement 1.

**Figure 1.**
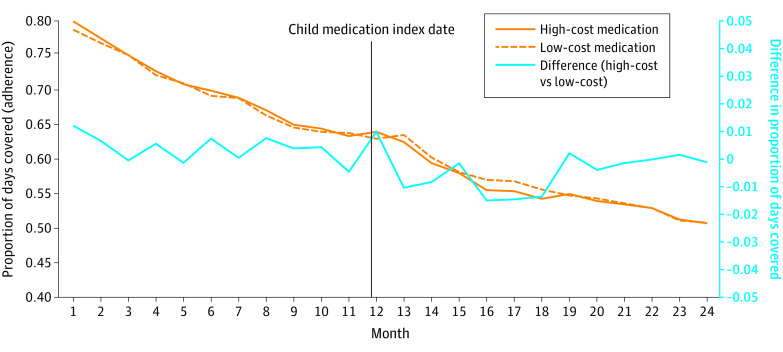
Trends in Parental Adherence to Long-Term Disease Medication by Cost: Long-Term Medication Initiation Monthly adherence to medication measured by the proportion of days covered in the high-cost and low-cost groups in the long-term matched cohort is plotted on the left y-axis before and after the child index date. The difference in adherence between groups is plotted on the right y-axis.

**Figure 2.**
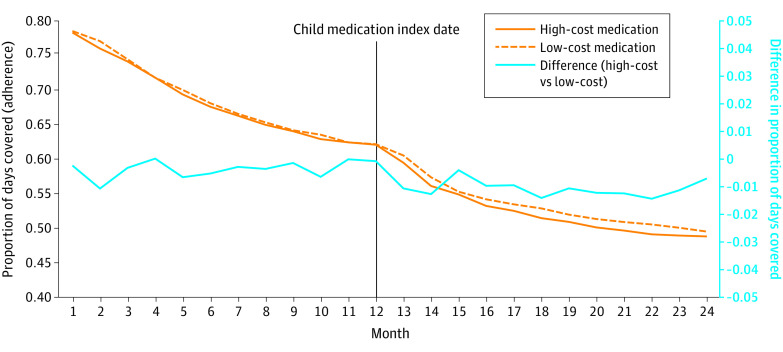
Trends in Parental Adherence to Long-Term Disease Medication by Cost: Short-Term Medication Initiation Monthly adherence to medication measured by the proportion of days covered in the high-cost and low-cost groups in the short-term matched cohort is plotted on the left y-axis before and after the child index date. The difference in adherence between groups is plotted on the right y-axis.

Time trends in parental adherence by whether their child initiated a high cost or low cost in the long-term and short-term cohorts are shown in Figure 1 and Figure 2. All groups experienced decreasing adherence trends over time, similar to typical adherence trends.^5,11,43^ The overall trends were similar at baseline between groups and both cohorts.

In interrupted time-series analyses (Table 2), compared with adherence among parents whose children started a low-cost long-term medication, those whose children initiated a high-cost long-term medication were associated with an immediate 1.9% (95% CI, −3.8% to −0.9%) and sustained (slope, 0.2%; 95% CI, −0.1% to 0.4%) absolute reduction in adherence for their parents. Similarly, the initiation of a chronic high-cost medication was associated with a 4.8% (95% CI, −8.6% to −1.0%) lower odds of optimal adherence and stayed lower over follow-up. In the acute cohort, the initiation of a high-cost medication was associated with a smaller immediate 0.6% (95% CI, −1.3% to −0.01%) absolute reduction, which also remained lower. Initiating a short-term high-cost medication was also associated with a 1.9% (95% CI, −3.3% to −0.6%) lower odds of optimal adherence staying lower over follow-up.

**Table 2.** Association Between High-Cost Child Medication Initiation and Parental Adherence to Long-Term Disease Medication

Measure	Parameter^a^	Change in adherence (95% CI), %
**Long-term cohort (highest vs lowest decile of child costs)**		
Adherence (PDC)	Change in level	−1.94% (−3.79% to −0.9%)
	Change in slope	0.16% (−0.08% to 0.41%)
Odds of being optimally adherent (PDC ≥80%)	Change in level	−4.81% (−8.59% to −1.02%)
	Change in slope	0.57% (0.03% to 1.11%)
**Short-term cohort (highest vs lowest decile of child costs)**		
Adherence (PDC)	Change in level	−0.63% (−1.25% to −0.01%)
	Change in slope	−0.02% (−0.10% to 0.07%)
Odds of being optimally adherent (PDC ≥80%)	Change in level	−1.93% (−3.26% to −0.59%)
	Change in slope	−0% (−0.19% to 0.19%)

Abbreviation: PDC, proportion of days covered.

^a^ Change in level corresponds to the immediate change; change in slope corresponds to the change in the subsequent trend.

Exploratory subgroups are shown in eFigures 2A and 2B in Supplement 1. The biggest modifiers were baseline levels of parent adherence, prior length of treatment, and family-level prior out-of-pocket medication spending. For instance, parents who first filled their medication 180 days or more prior to their child starting a long-term medication experienced an immediate 2.7% (95% CI, −4.7% to −0.7%) absolute adherence reduction, whereas those starting treatment in the past 180 days experienced no significant drop. Previously-adherent parents experienced a slightly greater immediate absolute drop (−2.8%; 95% CI, −4.9% to −0.6%) vs those who were nonadherent (1.0%; 95% CI, −4.0% to 1.9%). Families who had spent more out-of-pocket on medications also appeared more susceptible to medication costs; parents in the top 25% of family out-of-pocket spending experienced a 3.8% (95% CI, −7.2% to −0.5%) absolute drop in adherence, which was more than those in the bottom 25% (−1.7%; 95% CI, −3.9% to 0.5%, interaction *P* = .005).

Results were similar when high cost and low cost were defined by quartiles and medians (eTable 4 in Supplement 1). Other sensitivity analyses evaluating differences in definitions and exposure time windows were also similar (eTable 5 in Supplement 1). The biggest difference from the primary analysis was that initiating a high-cost medication was associated with a greater immediate reduction in parental adherence when the exposure window was extended to include the entire first 90 days of spending to define high cost and low cost vs just the index date (ie, immediate reduction for the long-term cohort, −3.0%; 95% CI, −4.4% to −1.5%).

## Discussion

High drug costs contribute to poor adherence^44^ with up to 25% of adults reporting cost-related medication underuse.^7^ Evaluations of cost-related medication nonadherence have focused on costs to the individual.^7^ However, many families share financial resources, and medication costs for a child could contribute to cost-related nonadherence for a parent.^12^ Although other work has observed relationships between poor adherence to medication within families,^14,15,45^ the specific association between high medication costs between children and their parents’ adherence had not previously been evaluated. In this interrupted time-series analysis, a child starting a high-cost medication was associated with a small immediate reduction in their presumed parent’s adherence, which persisted over time. This association was larger for medications intended for long-term use but also occurred for short-term use.

Reductions in adherence of the magnitude we observed, especially those in some exploratory subgroups (ie, approximately 3%-5% reductions in adherence) have been associated with worse patient outcomes and higher health care spending in some observational studies and a prior randomized trial.^30,46,47^ Parents who were previously adherent or established in therapy, and families who spent more out-of-pocket on medications appeared to be more sensitive. This cost sensitivity could be explained by greater underlying sickness or some regression to the mean (although we would expect this to be nondifferential). In addition, evaluating costs over a longer period (ie, 90 days) was also associated with worse parental adherence. Because differences persisted over time, this lower adherence would translate to an estimated 20 fewer days with medication over a 1-year follow-up.

The specific reason for the potential relationship between child medication costs and parental adherence is worth considering. Higher prescription costs themselves could be the primary mechanism because this was the key exposure we studied after adjusting for all other measured factors, and this relationship on the individual has been extensively uncovered.^7,44,48^ Because the patients in this study were all covered by the same insurer, the payer may be less relevant than in other settings, for example, those with public insurance who may experience greater financial hardship.^44^ The out-of-pocket costs themselves were also somewhat modest, which suggests a high level of cost sensitivity, even within this population. However, it is also possible that managing a new medication (and possibly, a new medical condition) in a child itself created a new stressor for the parent and led to a disruption in managing their own health, which the more expensive medication could have been a proxy for. This is consistent with prior research demonstrating that clinical events in one family member are negatively associated with adherence in another.^14,15^

These findings have several clinical and policy implications. First, these results continue to highlight that more should be done to decrease out-of-pocket prescription costs,^49^ for example to consider that spending by dependents is also considered. Prior research has shown that interventions to decrease patient-sharing costs improve medication adherence and clinical outcomes.^5,48,50^ Particularly concerning was that parents who already spent more on prescriptions were more likely to be affected. Thus, expanding policy initiatives to decrease out-of-pocket medication costs for families may be even more important, especially because spending on medications has been shown to offset total health care spending, even in children.^51^ Second, that the negative association between high costs and parents who were previously adherent was greater may be important for adherence interventions. Interventions could be designed to target families rather than individuals, or be delivered at different points of care, such as during clinical visits for their children. Finally, when reporting out-of-pocket spending and the effects of medication costs, investigators should consider reporting on the family level because these types of identifiers are often available in claims data. Regardless, the prevalence of medication use and health care costs in children and adolescents is growing^20,22^; more research is necessary to elucidate the relationship between medication costs and adherence among family members, related risk factors, and consequences, including research on how family members make tradeoffs.

### Limitations

This study has several limitations. First, we used claims data, so some bias is possible due to incompletely measured confounders. Second, adherence was measured using days’ supply; althoughvalidated and commonly-used, claims data may overestimate or underestimate adherence, but should be nondifferential.^52,53,54^ Third, a child had to fill a medication to be included, which may underestimate the association because prescriptions that were written and never filled are not captured.^55^ Thus, we likely actually underestimated the potential relationship between drug costs within families; we focused on only 1 child and parent to ensure fair comparisons, but other family members may also contribute to cost-related nonadherence. Fourth, we could not determine the exact nature of the relationship of the adult (ie, parent or much older sibling) but this is unlikely to be differential and they were at least in the same insured family unit. Fifth, individual-level income (and household expenditures) is also unavailable from claims; associations may be even greater if low-income families could have been specifically studied because their cost sensitivity is often higher.^7,44^ The data may also not be generalizable to Medicaid, which typically has lower copayments. Finally, the subgroup analyses were exploratory.

## Conclusions

This cohort study found that costs of a new medication by a child may have a small but meaningful immediate association with reductions in their parent’s adherence to long-term medications. Interventions aimed at improving adherence and health outcomes should potentially broaden to consider the effects of children or even household-level spending rather than individual-level spending alone.

## Notes

Supplement 1. **eFigure 1.** Schematic of cohort creation **eTable 1.** Number of patients and study eligibility after applying criteria **eTable 2.** Baseline characteristics for high-cost and low-cost child-parent pairs before matching **eFigure 2A.** Subgroups comparing high-cost child medication initiation and parental adherence to chronic disease medication: Chronic cohort **eFigure 2B.** Subgroups comparing high-cost child medication initiation and parental adherence to chronic disease medication: Acute cohort **eTable 3.** Medication costs by parents in the baseline period prior to the child index date **eTable 4.** Association between high-cost child medication initiation and parental adherence to chronic disease medications: by upper versus lower quartiles and above and below the median **eTable 5.** Sensitivity analyses of the primary outcome

Supplement 2.
Data Sharing Statement
