# Supplementary material for: Costs of Prescription Drugs for Children and Parental Adherence to Long-Term Medications
Source: JAMA Netw Open. 2023 Oct 16;6(10):e2337971. doi: 10.1001/jamanetworkopen.2023.37971 (PMC10580109; doi:10.1001/jamanetworkopen.2023.37971)
Supplement: Supplement 1. — eFigure 1. Schematic of cohort creation eTable 1. Number of patients and study eligibility after applying criteria eTable 2. Baseline characteristics for high-cost and low-cost child-parent pairs before matching eFigure 2A. Subgroups comparing high-cost child medication initiation and parental adherence to chronic disease medication: Chronic cohort eFigure 2B. Subgroups comparing high-cost child medication initiation and parental adherence to chronic disease medication: Acute cohort eTable 3. Medication costs by parents in the baseline period prior to the child index date eTable 4. Association between high-cost child medication initiation and parental adherence to chronic disease medications: by upper versus lower quartiles and above and below the median eTable 5. Sensitivity analyses of the primary outcome [file jamanetwopen-e2337971-s001.pdf]

Supplemental Online Content

Lauffenburger JC, Barlev RA, Olatunji E, Brill G, Choudhry NK. Costs of prescription drugs for children and parental adherence to long-term medications. *JAMA Network Open*. 2023;6(10):e2337971.
doi:10.1001/jamanetworkopen.2023.37971

eFigure 1. Schematic of cohort creation
eTable 1. Number of patients and study eligibility after applying criteria
eTable 2. Baseline characteristics for high-cost and low-cost child-parent pairs before matching
eFigure 2A. Subgroups comparing high-cost child medication initiation and parental adherence to chronic disease medication: Chronic cohort
eFigure 2B. Subgroups comparing high-cost child medication initiation and parental adherence to chronic disease medication: Acute cohort
eTable 3. Medication costs by parents in the baseline period prior to the child index date
eTable 4. Association between high-cost child medication initiation and parental adherence to chronic disease medications: by upper versus lower quartiles and above and below the median
eTable 5. Sensitivity analyses of the primary outcome

This supplemental material has been provided by the authors to give readers additional information about their work.

© 2023 Lauffenburger JC et al. *JAMA Network Open*

eFigure 1. Schematic of cohort creation

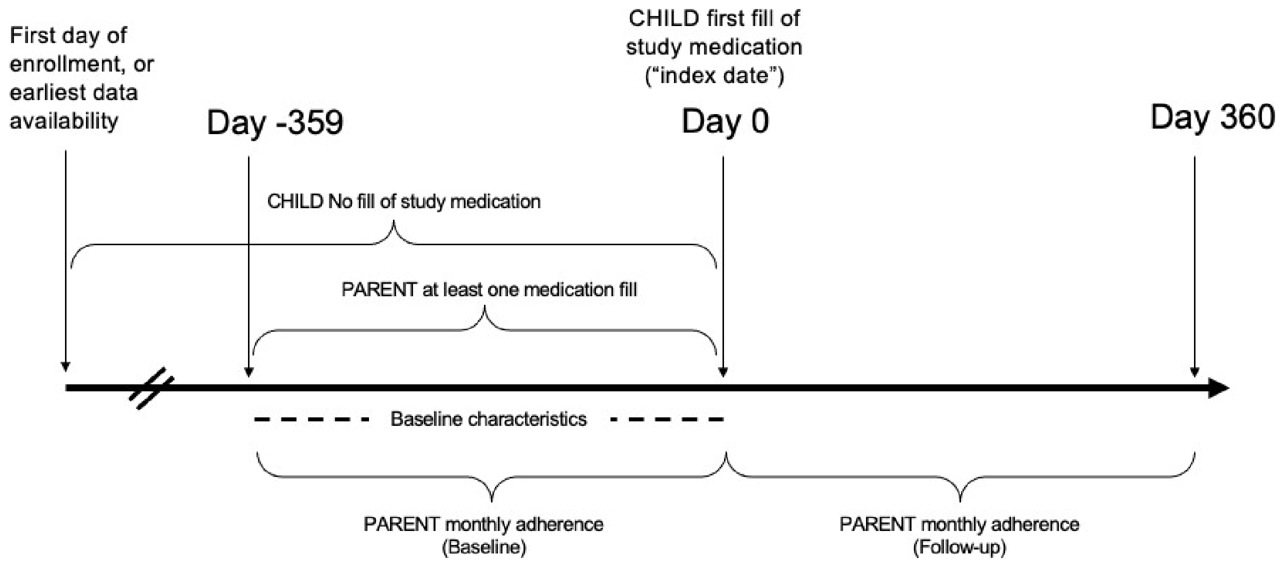

© 2023 Lauffenburger JC et al. *JAMA Network Open*

eTable 1. Number of patients and study eligibility after applying criteria

Study Criteria	Number of children remaining	Number of parents remaining
<18 years of age, initiating a medication on the Top 200 child drug list	1,466,768	-
Continuous enrollment ≥365 days before index date for child/adolescent <18 years of age	1,128,283	-
Have ≥1 adult family member (≥15 years older and ≥18 years of age) within family unit who previously filled a medication on the Top 200 adult drug list	310,267	359,580
Continuous enrollment ≥365 days before index date for ≥1 adult family member	310,267	354,002
First eligible child within family unit	309,682	354,002
First eligible adult family member within family unit	309,682 (n=309,682 children, n=309,682 parents)<br>Eligible child-parent pair – labeled as acute or chronic cohort	
Top or bottom decile of child medication costs (assessed separately for acute or chronic cohorts)	60,299 child-parent pairs	
Propensity score matching (including matching on baseline trends)	4,774 child-parent pairs in chronic cohort (61.5% matched)<br>42,380 child-parent pairs in acute cohort (80.5% matched)	

© 2023 Lauffenburger JC et al. *JAMA Network Open*

eTable 2. Baseline characteristics for high-cost and low-cost child-parent pairs before matching

	Chronic Cohort		Acute Cohort	
	Low Decile (n=4,471)	High Decile (n=3,292)	Low Decile (n =27,720)	High Decile (n =24,816)
**CHILD**				
Age, mean (SD)	13.2 (4.7)	12.6 (3.6)	10.2 (5.3)	9.6 (4.8)
Female, N (%)	3,539 (79.2)	1,558 (47.3)	13,603 (49.1)	12,663 (51.0)
Mail order for study medication, N (%)	27 (0.6)	56 (1.7)	9 (0.0)	54 (0.2)
Unique number of medications, mean (SD)	1.20 (0.58)	1.28 (0.66)	1.41 (0.72)	1.27 (0.58)
**PARENT**				
Age, mean (SD)	44.8 (7.5)	45.2 (7.3)	42.6 (7.8)	42.5 (7.6)
Female, N (%)	2,199 (49.2)	1,630 (49.5)	14,154 (51.1)	12,952 (52.2)
Race/Ethnicity, N (%)				
Asian	181 (4.0)	176 (5.3)	1,761 (6.4)	1,780 (7.2)
Black, Non-Hispanic	306 (6.8)	279 (8.5)	2,164 (7.8)	1,913 (7.7)
Hispanic	367 (8.2)	292 (8.9)	3,092 (11.2)	2,656 (10.7)
White	3,142 (70.3)	2,269 (68.9)	17,921 (64.7)	16,404 (66.1)
Unknown	475 (10.6)	276 (8.4)	2,782 (10.0)	2,063 (8.3)
Region, N (%)				
Northeast	367 (8.2)	195 (5.9)	1,589 (5.7)	1,642 (6.6)
Midwest	1,092 (24.4)	585 (17.8)	6,131 (22.1)	4,029 (16.2)
South	930 (20.8)	934 (28.4)	6,828 (24.6)	8,062 (32.5)
West	723 (16.2)	431 (13.1)	4,120 (14.9)	2,958 (11.9)
Unknown	1,359 (30.4)	1,147 (34.8)	9,052 (32.7)	8,125 (32.7)
**Medication characteristics**				
Mail order for study medication, N (%)	198 (4.4)	181 (5.5)	1,269 (4.6)	1,220 (4.9)
Brand name for study medication, N (%)	242 (5.4)	319 (9.7)	1,915 (6.9)	2,293 (9.2)
**Health resource utilization, mean (SD)**				
Unique number of medications	6.2 (4.9)	5.4 (4.1)	6.4 (4.7)	5.6 (4.0)
Number of physician office visits	5.4 (5.1)	4.5 (4.1)	5.5 (5.2)	4.8 (4.3)
Number of days hospitalized	0.6 (4.1)	0.3 (2.2)	0.7 (4.6)	0.3 (2.4)
**Comorbidities**				
Combined comorbidity score, mean (SD)	0.3 (1.3)	0.1 (1.0)	0.3 (1.3)	0.2 (1.1)
Acquired Hypothyroidism, N (%)	499 (11.2)	330 (10.0)	3,379 (12.2)	2,928 (11.8)
Asthma, N (%)	201 (4.5)	110 (3.3)	1,235 (4.5)	961 (3.9)
Atrial Fibrillation, N (%)	42 (0.9)	26 (0.8)	260 (0.9)	174 (0.7)
Chronic Kidney Disease, N (%)	199 (4.5)	74 (2.2)	1,257 (4.5)	690 (2.8)
COPD, N (%)	50 (1.1)	22 (0.7)	259 (0.9)	149 (0.6)
Depression, N (%)	711 (15.9)	387 (11.8)	3,886 (14.0)	2,760 (11.1)
Diabetes, N (%)	495 (11.1)	275 (8.4)	2,936 (10.6)	2,190 (8.8)
Epilepsy, N (%)	29 (0.6)	17 (0.5)	175 (0.6)	128 (0.5)
Heart Failure, N (%)	46 (1.0)	33 (1.0)	331 (1.2)	192 (0.8)
Hyperlipidemia, N (%)	911 (20.4)	686 (20.8)	5,675 (20.5)	5,018 (20.2)
Hypertension, N (%)	1,212 (27.1)	799 (24.3)	7,192 (25.9)	5,676 (22.9)
Ischemic Heart Disease, N (%)	196 (4.4)	125 (3.8)	1,047 (3.8)	764 (3.1)
Liver Disease, N (%)	73 (1.6)	30 (0.9)	527 (1.9)	284 (1.1)
Stroke/Transient Ischemic Attack, N (%)	58 (1.3)	28 (0.9)	324 (1.2)	195 (0.8)
**FAMILY UNIT**				
Total number of children, mean (SD)	2.0 (1.1)	2.0 (1.0)	2.2 (1.1)	2.1 (1.0)
Total family OOP pharmacy costs[§], in $	605.0 (993.2)	790.5 (931.6)	582.9 (937.1)	627.9 (742.6)
Total family OOP medical costs, in $	6794.9 (7759.2)	5386.8 (5802.4)	7245.5 (8045.5)	5163.9 (6188.7)

[§]Excluding cost of study drug
Abbreviations: IQR, Interquartile range; ASD, Absolute standardized difference; SD, Standard deviation

© 2023 Lauffenburger JC et al. *JAMA Network Open*

eFigure 2.

A. Subgroups comparing high-cost child medication initiation and parental adherence to chronic disease medication: Chronic cohort

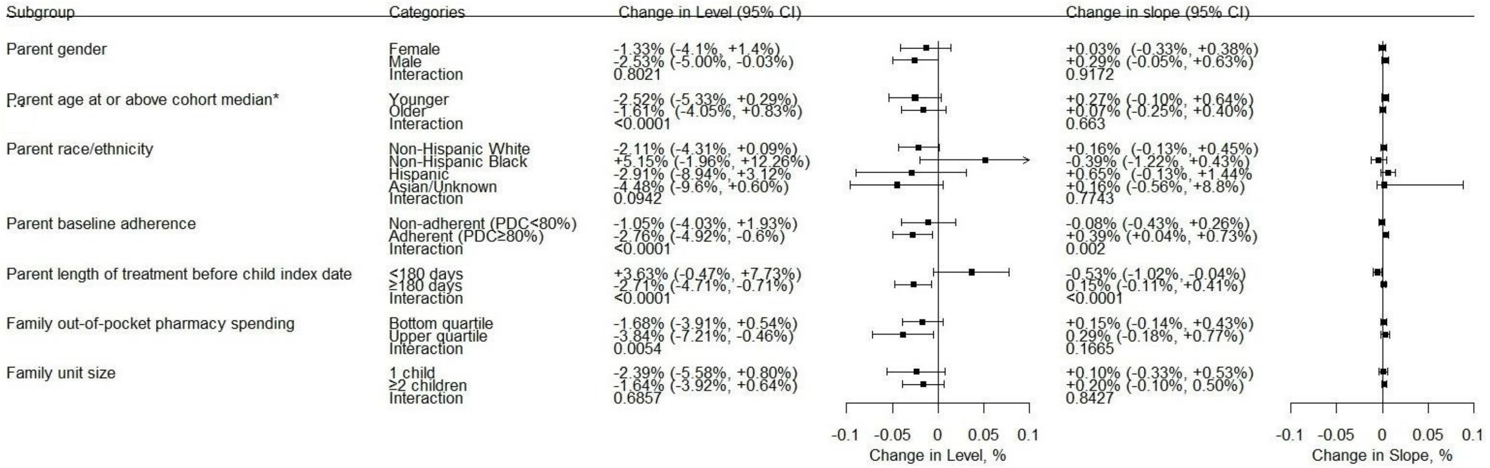

B. Subgroups comparing high-cost child medication initiation and parental adherence to chronic disease medication: Acute cohort

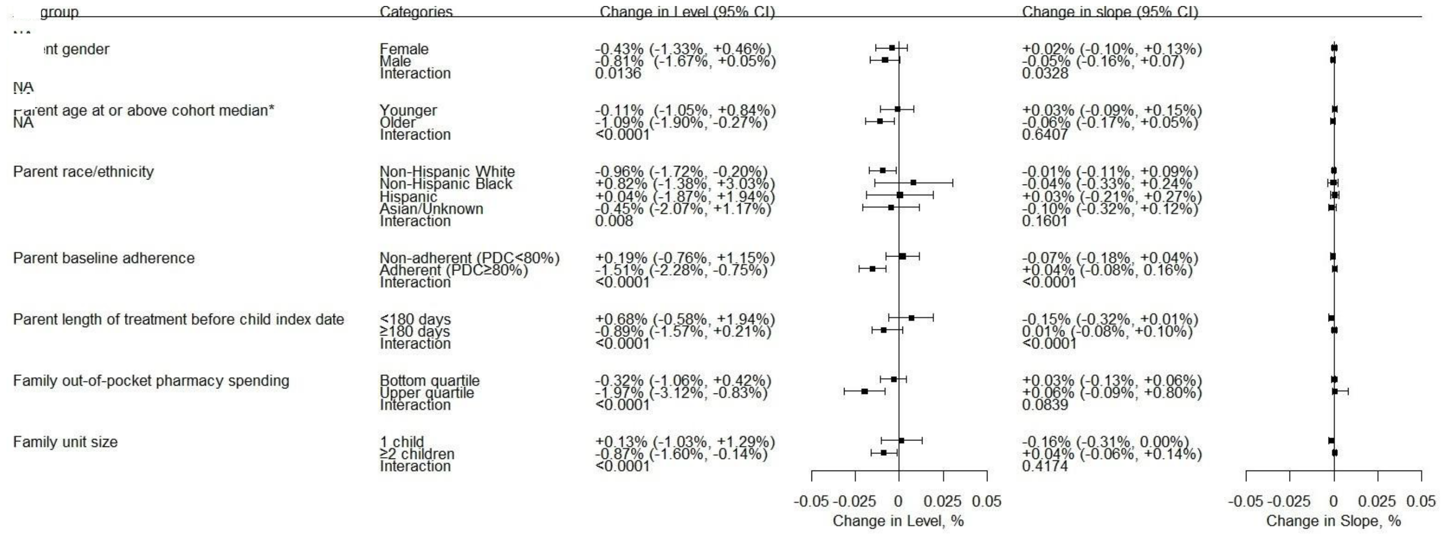

Footnote:
*Chronic median age: 45; acute median age: 42
Abbreviations: CI, Confidence interval; PDC, proportion of days covered

© 2023 Lauffenburger JC et al. *JAMA Network Open*

eTable 3. Medication costs by parents in the baseline period prior to the child index date

Cohort	Median (IQR) of out-of-pocket spending measured across the parents' baseline period, $
Chronic: Low-cost Decile	590.00 (270.00-1257.00)
Chronic: High-cost Decile	523.00 (245.50-1027.50)
Acute: Low-cost Decile	560.00 (255.50-1179.00)
Acute: High-cost Decile	497.50 (235.00-1020.00)

Abbreviations: IQR, Interquartile range

© 2023 Lauffenburger JC et al. *JAMA Network Open*

eTable 4. Association between high-cost child medication initiation and parental adherence to chronic disease medications: by upper versus lower quartiles and above and below the median

Monthly Measure	Parameter	Change in adherence (95% CI)
**CHRONIC COHORT (highest vs. lowest quartile of child costs)**		
Adherence (PDC)	Change in level	-1.21% (-2.35%, -0.07%)
	Change in slope	0.08% (-0.08%, 0.23%)
Odds of being fully adherent (PDC≥0.80)	Change in level	-2.85% (-5.20%, -0.49%)
	Change in slope	0.21% (-0.14%, 0.55%)
**ACUTE COHORT (highest vs. lowest quartile of child costs)**		
Adherence (PDC)	Change in level	-0.38% (-0.77%, 0.01%)
	Change in slope	-0.04% (-0.09%, 0.01%)
Odds of being fully adherent (PDC≥0.80)	Change in level	-1.13% (-1.99%, -0.27%)
	Change in slope	-0.09% (-0.21%, 0.03%)
**CHRONIC COHORT (above vs. below median of child costs)**		
Adherence (PDC)	Change in level	-1.01% (-2.15%, 0.13%)
	Change in slope	0.05% (-0.07%, 0.17%)
Odds of being fully adherent (PDC≥0.80)	Change in level	-1.75% (-3.32%, -0.18%)
	Change in slope	0.13% (-0.11%, 0.37%)
**ACUTE COHORT (above vs. below median of child costs)**		
Adherence (PDC)	Change in level	-0.22% (-0.54%, 0.10%)
	Change in slope	-0.02% (-0.06%, 0.02%)
Odds of being fully adherent (PDC≥0.80)	Change in level	-0.76% (-1.30%, -0.22%)
	Change in slope	-0.05% (-0.16%, 0.06%)

Abbreviations: CI, Confidence interval; PDC, proportion of days covered

eTable 5. Sensitivity analyses of the primary outcome

Adherence (PDC)	Parameter	Change in adherence (95% CI)
**CHRONIC COHORT (highest vs. lowest decile of child costs)**		
Original	Change in level	-1.94% (-3.79%, -0.9%)
	Change in slope	+0.16% (-0.08%, +0.41%)
Pairs with ≥20 years age difference	Change in level	-1.95% (-3.83%, -0.08%)
	Change in slope	-0.18% (-0.07%, +0.42%)
90-day transition period	Change in level	-2.01% (-4.03%, -0.02%)
	Change in slope	+0.08% (-0.10%, +0.31%)
Extended exposure window	Change in level	-2.98% (-4.42%, -1.54%)
	Change in slope	+0.08% (-0.13%, +0.29%)
**ACUTE COHORT (highest vs. lowest decile of child costs)**		
Original	Change in level	-0.63% (-1.25%, -0.01%)
	Change in slope	-0.02% (-0.10%, +0.07%)
Pairs with ≥20 years age difference	Change in level	-0.70% (-1.33%, -0.08%)
	Change in slope	-0.01% (-0.10%, +0.07%)
90-day transition period	Change in level	-0.73% (-1.04%, -0.43%)
	Change in slope	-0.07% (-0.15%, +0.01%)
Extended exposure window	Change in level	-1.19% (-1.76%, -0.63%)
	Change in slope	+0.02% (-0.11%, +0.08%)

© 2023 Lauffenburger JC et al. *JAMA Network Open*
