# Supplementary material for: Costs of Prescription Drugs for Children and Parental Adherence to Long-Term Medications
Source: JAMA Netw Open. 2023 Oct 16;6(10):e2337971. doi: 10.1001/jamanetworkopen.2023.37971 (PMC10580109; doi:10.1001/jamanetworkopen.2023.37971)
Supplement: Supplement 2. — Data Sharing Statement [file jamanetwopen-e2337971-s002.pdf]

Data Sharing Statement

Data
Data available: Yes
Data types: Deidentified participant data
How to access data: Patient data will be available upon request, pending appropriate data use agreements executed.
When available: With publication

Supporting Documents
Document types: Statistical/analytic code
How to access documents: Code available upon request by contacting the corresponding author.
When available: With publication

Additional Information
Who can access the data: Researchers whose proposed use of the data has been approved
Types of analyses: For research purposes
Mechanisms of data availability: With a signed data access agreement and other necessary agreements
